# Discovery of Lactoferrin as a Stimulant for hADSC-Derived EV Secretion and Proof of Enhancement of Resulting EVs through Skin Model

**DOI:** 10.3390/ijms222010993

**Published:** 2021-10-12

**Authors:** Junho Kim, Ga Eun You, Minkyu Woo, Nicole Hyesoo Chang, Jungsun Lee

**Affiliations:** Research and Development Institute, Biosolution Co., Ltd., Seoul Technopark, 232 Gongneung-ro, Nowon-gu, Seoul 01811, Korea; gerocount@biosolutions.co.kr (J.K.); yougaeun88@biosolutions.co.kr (G.E.Y.); arcus913@biosolutions.co.kr (M.W.); nchang@biosolutions.co.kr (N.H.C.)

**Keywords:** extracellular vesicles, exosome, human lactoferrin, stem cell, calcium regulation, stimulant

## Abstract

Extracellular vesicles (EVs) are secreted from hADSCs in low concentrations, which makes it difficult to utilize them for the development of therapeutic products. To overcome the problem associated with low concentration, we proposed human lactoferrin (hLF) as a stimulant for the secretion of hADSC-derived EVs. hLF has been reported to upregulate intracellular Ca^2+^, which is known to be capable of increasing EV secretion. We cultured hADSCs in hLF-supplemented media and analyzed the changes in intracellular Ca^2+^ concentration. The characteristics of hADSC-derived EVs secreted by hLF stimulation were analyzed through their number, membrane protein markers, and the presence of hLFs to EVs. The function of hADSC-derived EVs was investigated through their effects on dermal fibroblasts. We found that hLF helped hADSCs effectively uptake Ca^2+^, resulting in an increase of EVs secretion by more than a factor of 4. The resulting EVs had enhanced proliferation and collagen synthesis effect on dermal fibroblasts when compared to the same number of hADSC-derived EVs secreted without hLF stimulation. The enhanced secretion of hADSC-derived EVs increased collagen synthesis through enhanced epidermal penetration, which resulted from increased EV numbers. In summary, we propose hLF to be a useful stimulant in increasing the secretion rate of hADSC-derived EVs.

## 1. Introduction

Stem cell-derived EVs contain RNAs, proteins, and lipids derived from stem cells, and are known as carriers that effectively deliver the therapeutic function of stem cells to other cells [1]. Therefore, stem cell-derived EVs have been gaining increased attention in the field of regeneration and immune modulation as a functional ingredient in therapeutic products [2,3,4]. Many reports have demonstrated stem-cell derived EV-mediated tissue regeneration in various organ systems, such as the nervous, cardiovascular, hepatic, respiratory, and renal systems [5]. Regenerative therapeutics based on stem cell-derived EVs have the advantage of reduced potential complications such as cancer formation and blood vessel occlusion, both of which are notable problems in pre-existing cell-based therapeutics [6]. Stem cell-derived EVs are also capable of movement through tissue barriers such as the epidermis and the blood–brain barrier [7,8,9]. This characteristic allows stem cell-derived EVs to exhibit regeneration effects in dermal fibroblasts and neurons [6,7,8,9,10,11]. 

However, the amount of EVs released from most cells is minute in its quantity, which hinders their application in practical use [12,13]. Studies have reported an increase in EV secretion rate where stimulants were added to the cells [14], such as external stress, cytoskeleton blocking, overexpression of EV components, and induced intracellular Ca^2+^ upregulation [15,16,17,18,19,20]. In general, the stimulus causes a functional change by altering the internal and/or surface composition of EVs, which has the potential to induce cytotoxicity in cells that generate or receive EVs [14]. Increasing the intracellular calcium level, for example, is a well-known method in increasing both the biogenesis of microvesicles and exosomes in cells. It induces EV generation by increasing intracellular secretory granules and remodeling the plasma membrane [21,22,23,24]. The Na^+^ ionophore has been reported as an intracellular Ca^2+^ upregulator that stimulates the generation of EVs [21,22]. However, Na^+^ ionophores can induce oxidative stress and inhibit stem cell proliferation through the Na^+^/H^+^ exchange process which may result in cellular apoptosis [25]. Therefore, the application of stimulants such as Na^+^ ionophores require more research for application into stem cell-derived EVs therapy. As such, the changes caused to stem cell-derived EVs by stimulants should be investigated in the context of whether or not they impede the applicable effects of their intended use.

hLF is a multi-functional protein that is present in large quantities in milk, and is known to confer beneficial effects against diseases, inflammation, and wounds [26,27]. Our team studied the beneficial effects of hLF as a stimulant for human stem cell-derived EV generation. First of all, hLF has a Fe^3+^-binding affinity and can transfer Fe^3+^ ions into cells. Therefore, hLF is used as a factor in human stem cell culture to increase the survival rate of stem cells via Fe^3+^ ion transfer. Secondly, it has recently been confirmed that hLF is involved in the activation of Ca^2+^ channels in human neurons and human neutrophils [28,29], which suggests that it may potentially enhance EV biogenesis from human stem cells. Finally, hLF is known to be effective in enhancing cell proliferation and ECM synthesis in target human cells [27,30,31]. Among studies of interest, some have conducted studies on delivering hLF through EVs by exploiting its binding affinity to specific receptors on the surface of EVs [32,33]. The delivery of hLF through EVs has the potential to improve the efficacy of EVs, especially where cell proliferation and extracellular matrix (ECM) synthesis is concerned. Due to the three reasons above, hLF is expected to have potential in providing an adequate culture condition conductive to the enhanced biogenesis of human stem cell-derived EVs. No study so far has reported the effect of hLF on the biogenesis of EVs from human stem cells; thus, in this study we assumed that hLF plays a role in enhancing both the generation and the function of stem cell-derived EVs.

The study was conducted with human adipose-derived stem cells (hADSC), one of the commonly used mesenchymal stem cells in regeneration therapeutics [34]. The purpose of this study was to confirm that hLF acts as a stimulant for the secretion of hADSC-derived EV and does not act as an inhibitor for the function of individual hADSC-derived EVs. The resulting functional changes of hADSC-derived EVs were investigated through their effect on dermal fibroblast activity. We demonstrated that hLF can increase the intracellular Ca^2+^ concentration in hADSCs, and consequently enhance the secretion of hADSC-EVs. Based on these results, we propose an effective hADSC culture condition which stimulates EV generation through hLF incorporation. In addition to stimulating EV biogenesis, hLF also has the property of binding to the newly generated hADSC-derived EVs during the stimulation process. In order to confirm that the hLFs bound to the hADSC-derived EVs do not impede the functionality of EVs, function changes of individual EVs were analyzed in terms of their effects on dermal fibroblast activity. In addition, to confirm that the EVs are suitable for enhanced tissue regeneration, they were applied to a 3D skin tissue model and observed for their efficacy. This novel cell culture method can be used as a foundational technology to overcome the relatively low productivity of hADSC-derived EV generation culture methods developed to date.

## 2. Results

### 2.1. Intracellular Calcium Regulation by hLF and EVs Secretion

**Figure 1:**
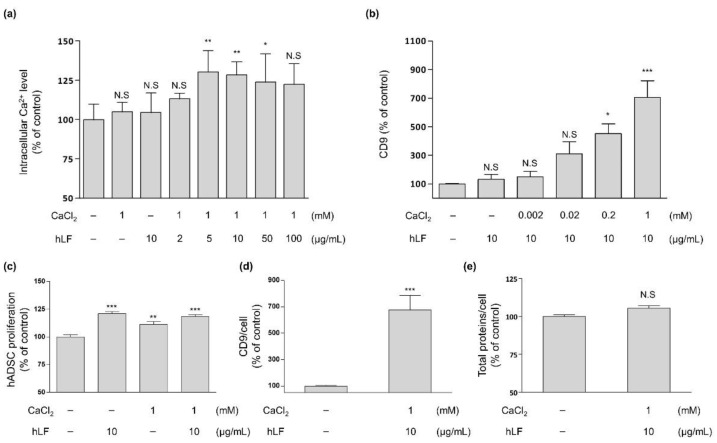
The relationship between intracellular Ca^2+^ level and CD9 secretion of hADSCs-stimulated by hLF. The hADSCs were cultured for 2 days in hLF-supplemented and/or calcium-supplemented basal media. (**a**) Intracellular Ca^2+^ levels were measured in lysed hADSCs (one-way ANOVA, *n* = 5, *p* = 0.0005). (**b**) CD9 secretion was measured in hADSC-CM. The addition of hLF at 10 µg/mL and CaCl_2_ at 1 mM to the media showed the highest increase in CD9 levels (one-way ANOVA, *n* ≥ 5, *p* < 0.0001). (**c**) The activity of hADSCs cultured for 2 days was investigated by MTT (3-[4,5 dimethylthioazole-2-yl]-2,5-diphenyl tetrazolium bromide) assay (one-way ANOVA, *n* = 6, *p* < 0.0001). (**a**–**c**) The significance between pairs was analyzed using post hoc, Tukey’s test. (**d**,**e**) The secretion data of CD9 and total protein was divided by the proliferation result of originating hADSCs (CD9: unpaired *t*-test, *n* ≥ 6, *p*= 0.0003; total protein: unpaired *t*-test, *n* = 3, *p*= 0.0596). All data were obtained respectively through at least three independent experimental groups and presented as the relative value compared to those of the non-supplemented basal media. Error bars represent ±SD. N.S (Not Significant) *p* > 0.05, * *p* < 0.05, ** *p* < 0.01, *** *p* < 0.001 were calculated versus the non-supplemented basal media.

We analyzed the concentrations of hLF effective in increasing the secretion of EVs from hADSCs. To investigate whether hLF influences the secretion of EVs through Ca^2+^ regulation in stem cells, we studied the relationship between hLF and intracellular Ca^2+^ levels in hADSCs. No significant differences in Ca^2+^ levels were found when either CaCl_2_ or hLF alone was added to the basal media. However, the addition of 5 µg/mL hLF to basal media supplemented by 1 mM of CaCl_2_ increased the intracellular Ca^2+^ levels in ADSCs by 30%, compared to hADSCs in basal media without the addition of hLF (Figure 1a).

We also examined whether the change in intracellular Ca^2+^ levels by hLF affected EV secretion. CD9, an EV membrane protein, is an EV-specific marker that can be directly quantified in the hADSC-CM and be used to distinguish EVs from other secreted soluble factors [35]. To this end, we investigated alterations in the CD9 concentrations of hADSC-CM by fixing the hLF concentration to a constant value of 10 µg/mL and varying CaCl_2_ concentration between 0 to 1 mM (Figure 1b). As a result, CD9 concentration was found to be increased in concordance with increasing CaCl_2_ concentration, with the highest peak observed at 1 mM of CaCl_2_ (approximately seven-fold increase in CD9 concentration). The addition of more than 1 mM of CaCl_2_ to the solution did not allow for further experimentation due to the precipitation of CaCl_2_ from the solution (data not shown). The combination of 10 µg/mL of lactoferrin and 1 mM of CaCl_2_ not only promoted the survival of hADSCs (Figure 1c), but also significantly increased the amount of CD9 expressed per hADSCs by 577% compared to control values without causing significant changes to total protein content levels (Figure 1d,e).

In conclusion, the increase in intracellular calcium levels induced by the addition of hLF to basal media containing Ca^2+^ is closely related to the production of EVs in hADSCs and did not induce cellular toxicity in hADSCs.

### 2.2. Productivity and Characteristic Change of EVs Generated from hLF-Stimulated hADSCs

**Figure 2:**
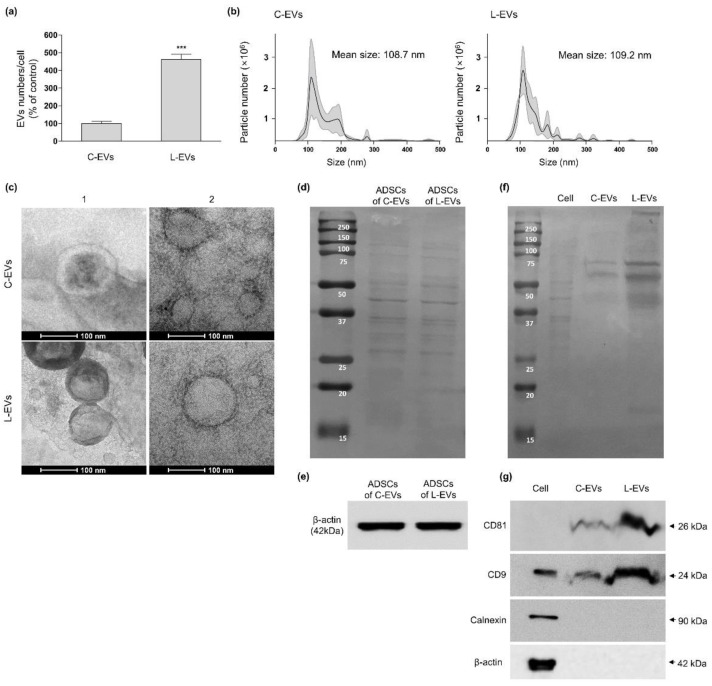
Productivity and morphologic characteristics of L-EVs and C-EVs. L-EVs and C-EVs were separated from their respective hADSC-CMs. (**a**,**b**) The number and size of EVs detected by NTA. (**a**) The number of EVs was divided by the number of originating hADSCs and presented as relative values compared to C-EVs (unpaired *t*-test, *n* = 5, *p* < 0.0001). Error bars represent ±SD, *** *p* < 0.001 were calculated versus the C-EVs. (**b**) Black solid lines: mean size of EVs; gray area: ±SD. (**c**) Representative TEM images of the EVs. Both C-EVs and L-EVs were negatively stained. Scale bars = 100 nm. (**d**) Same numbers of ADSCs were cultured in media containing 10 µg/mL of hLF and 1 mM of CaCl_2_ (ADSCs of L-EVs) or in media with none of the aforementioned additives (ADSCs of C-EVs), respectively. The result was obtained by ponceau S stain of ADSCs. (**e**) β-actin level of ADSCs detected by western blot. (**f**) Ponceau S stain of EVs. (**g**) CD81, CD9, calnexin, and β-actin levels of EVs were measured by western blot. The markers and protein compositions were measured in C-EVs and L-EVs lysates derived from the same number of hADSC. The results for CD81 and CD9 were obtained individually through two independent experiments. For calnexin, hADSC were used for positive control.

We defined hADSC-EVs generated with media containing 10 µg/mL of hLF and 1 mM of CaCl_2_ as “L-EV” and distinguished it from “C-EV”, which was prepared with none of the aforementioned additives. Our results confirmed that the number of L-EVs released per cell increased significantly by approximately 362% compared to C-EVs numbers (Figure 2a). C-EVs and L-EVs did not differ in terms of their average size (Figure 2b), and displayed similar morphology when investigated via transmission electron microscopy (TEM), characterized by a cup-shaped or spherical shaped lipid bilayer vesicle (Figure 2c).

EV positive and negative markers were used to determine the EV production rate per cell under C-EVs and L-EV conditions. The composition, amount of protein, β-actin extracted from hADSCs cultured in 10 µg/mL hLF and 1 mM CaCl_2_ did not show a significant difference in band compared to those of ADSCs generated with none of the aforementioned additives (Figure 2d,e). At the same time, the composition of C-EVs and L-EVs originated from the same number of ADSCs appeared to be similar, but L-EVs showed a stronger signal in terms of overall band intensity (Figure 2f). In western blot results for positive EV markers, CD9 and CD81 bands were shown to be clearly increased in L-EVs compared to C-EVs, whereas calnexin expression, an indicator of the endoplasmic reticulum, was undetectable in both EVs compared to hADSCs (Figure 2g). Collectively, our results indicate that basal media with an hLF concentration of 10 µg/mL and supplemented with 1 mM of CaCl_2_ enhances EV generation in hADSC, without the generation of apoptotic vesicles containing endoplasmic reticulum components.

**Figure 3:**
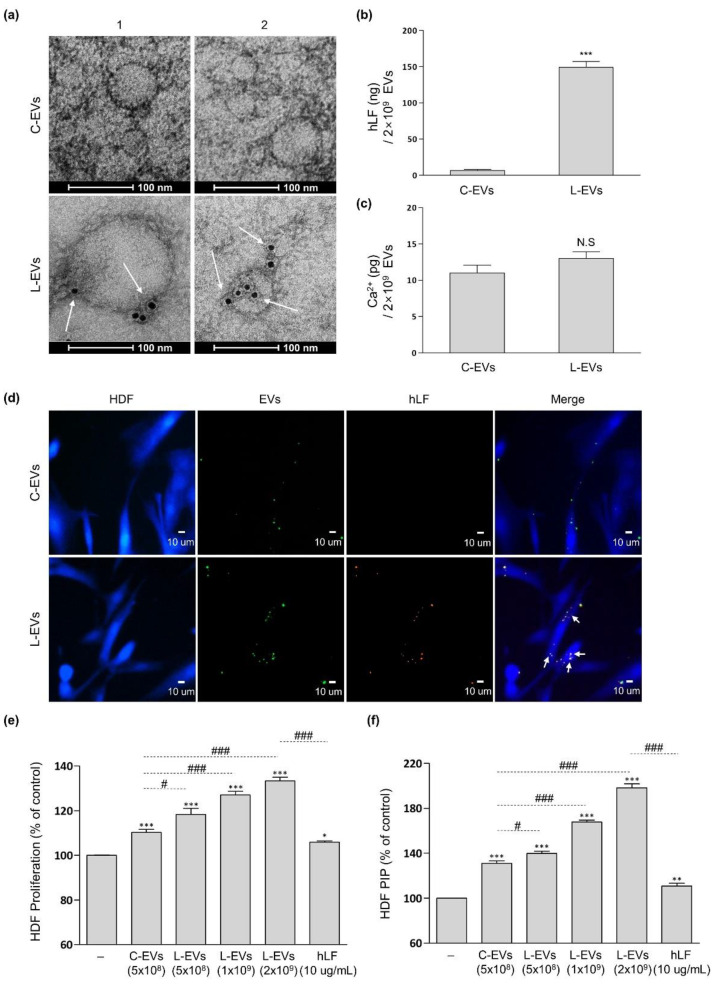
Binding of hLF to L-EVs and functional change of L-EVs. L-EVs and C-EVs were respectively separated from their hADSC-CMs. (**a**) TEM images showed hLF bound to the surface of L-EVs. The white arrows indicate anti-lactoferrin gold nanoparticles bound to L-EVs. Scale bars = 100 nm. (**b**,**c**) The amount of hLF and Ca^2+^ contained in the L-EVs was compared to those contained in C-EVs (hLF: unpaired *t*-test, *n* = 4, *p* < 0.0001; Ca^2+^: unpaired *t*-test, *n* = 4, *p* = 0.1035). The data were obtained through two independent experimental groups. Error bars represent ±SD. *** *p* < 0.001, N.S (not significant) *p* > 0.05 were calculated versus the C-EVs. (**d**) Image of hLF moving to HDFs via L-EVs. 5 × 10^7^ of DiO-stained EVs were used for both L-EVs and C-EVs. The white arrows indicate EVs stained simultaneously with anti-lactoferrin antibody (red) and DiO (green). HDF were stained by CellTracker (blue). Scale bars = 10 µm. (**e**,**f**) HDF proliferation and pro-collagen synthesis by C-EVs or L-EVs evaluated in terms of their number. The data were investigated using the MTT assay or PIP-ELISA after EVs or hLF were applied for 2 days. The data were obtained through three independent experimental groups (proliferation: one-way ANOVA, *n* ≥ 6, *p* < 0.0001; pro-collagen: one-way ANOVA, *n* ≥ 6, *p* < 0.0001) and significance between pairs was analyzed using post hoc, Tukey’s test. Error bars represent ±SD. * *p* < 0.05, ** *p* < 0.01, *** *p* < 0.001 were calculated versus the negative control. In the case of pairs connected by the dotted line, the difference between them was noted as # *p* < 0.05, ### *p* <0.001.

On the other hand, the hLF stimulated, hADSC-derived EVs were analyzed for changes in their characteristics. EVs are known to have receptors that hLFs can bind to [32,33]. As shown in Figure 3a, gold nanoparticles conjugated with the anti-lactoferrin antibody were found selectively on the membranes of L-EVs but not on those of C-EVs. The amount of hLF bound to L-EVs was in about 150 ng per 2 × 10^9^ L-EVs (Figure 3b). The amount of Ca^2+^ contained in L-EVs did not show a clear difference from that contained in C-EVs (Figure 3c). Thus, the possible transfer of hLF to cells via L-EVs was investigated. After staining the hLFs and the L-EVs membrane respectively, the L-EVs were applied to Human dermal fibroblasts (HDFs). As a result, it was confirmed that L-EVs and hLF do indeed move together (Figure 3d). These results suggest that the hLF used for hADSC culture could bind to L-EVs and that the binding between hLFs and L-EVs was not removed in the EVs separation process accompanied by washing, but was maintained throughout the process of moving L-EVs to HDFs.

To investigate the functional change of hADSC-derived EVs produced with hLF stimulation, the L-EVs were applied to HDFs in the same number as C-EVs or more, and then the effect of each respective EVs on HDF proliferation and collagen synthesis was analyzed (Figure 3e,f). As a result, when cell proliferation was compared between the same number of C-EVs and L-EVs at 5 × 10^8^ EVs, L-EVs showed an enhanced proliferative efficacy compared to C-EVs (C-EVs 10% vs. L-EVs 18%). When the number of L-EVs was increased to 1 × 10^9^ and 2× 10^9^, the proliferation of HDFs increased by 27% and 33%, respectively. The L-EVs were more effective than C-EVs in their ability to improve collagen synthesis at 5 × 10^8^ EVs (C-EVs 31% vs. L-EVs 40%). Collagen synthesis efficacy at 2 × 10^9^ L-EVs had increased by 98% compared to the control levels. Application of 10 µg/mL hLF to HDF by itself resulted in an increase of proliferation rate by 6%, and an increase in collagen synthesis by 11%. These values were significantly lower than the increases caused by 2× 10^9^ L-EVs to which in about 150 ng of hLF are bound to. These results prove that L-EVs are superior to C-EVs in terms of both HDF proliferation and ECM synthesis enhancement, and these effects were further increased when the number of L-EVs was increased.

### 2.3. L-EVs Generated through hLF Are Effective for ECM Synthesis of Skin Tissue Model

**Figure 4:**
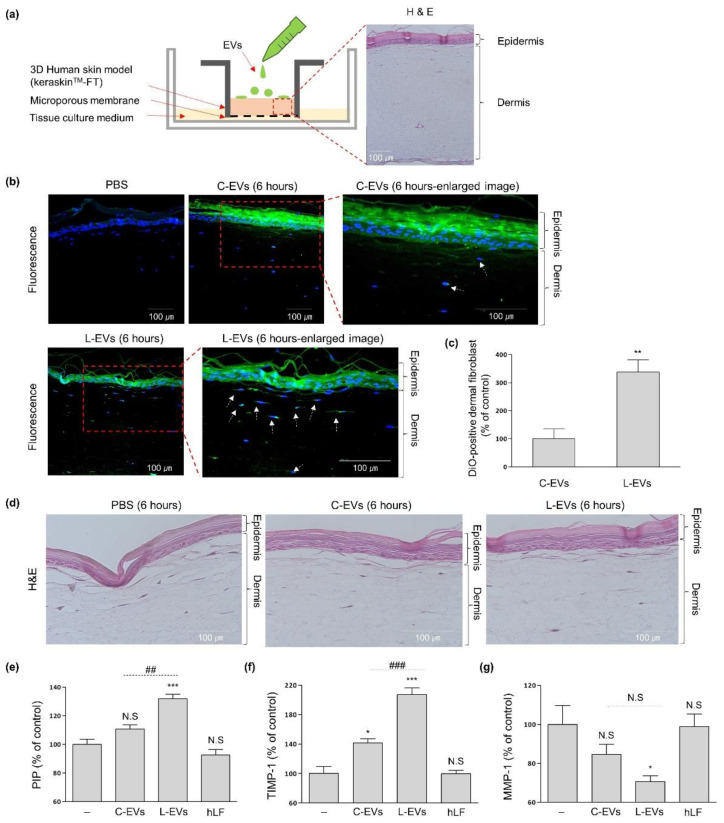
The effect of L-EVs permeability and ECM synthesis on skin tissue. L-EVs and C-EVs were derived from the same number of hADSC respectively. (**a**) Schematic diagram of EVs permeation in KeraSkin^TM^-FT and H&E cross-section image of skin model. Scale bars = 100 µm. (**b**) Human 3D skin models were treated with DiO-labeled EVs for 6 h. Scale bars = 100 µm. The permeation of EVs in skin tissue was investigated by a cross-section of the tissue. In 6 h enlarged image, the maximum fluorescence of L-EVs was found in the upper layer of the dermis. The white arrows indicate DiO-positive cells. (**c**) After 6 h treatment of EVs, DiO and DAPI-double positive cells of the dermis layer were counted at three independent cross-section of tissue (unpaired *t*-test, *n* = 3, *p* = 0.0064). (**d**) H&E cross-section image after treatment of EVs for 6 h. (**e**–**g**) After EVs or hLF (10 µg/mL) were treated for 48 h, the amount of PIP, TIMP-1, and MMP-1 secreted from the KeraSkin^TM^-FT into the media (tissue culture media) was collected and analyzed. (**c**,**e**–**g**) The value of the negative control was considered to be 100%, while those of the other groups were calculated as relative values. The PIP, TIMP-1, and MMP-1 values were statically analyzed using one-way ANOVA (PIP: *n* > 3, *p* < 0.0001; TIMP-1: *n* = 3, *p* < 0.0001; MMP-1: *n* = 3, *p* = 0.0382). The data were obtained through three independent experimental groups and significance between pairs were analyzed using post hoc, Tukey’s test. Error bars represent ±SD. * *p* < 0.05, ** *p* < 0.01, *** *p* < 0.001 were calculated versus the negative control. In the case of the pair connected by the dotted line, the difference between them was noted as ^##^ *p* < 0.01, ^###^ *p* < 0.001, N.S (Not Significant) *p* > 0.05.

Experiments were conducted to determine whether the enhanced secretion of L-EVs by hLF was also effective in enhancing the permeation of EVs through the skin’s epidermal barrier. L-EVs and C-EVs derived from the same number of hADSCs were stained with DiO. The DiO-stained EVs were topically applied to KeraSkin^TM^-FT (a reconstructed 3D-skin tissue model) for 6 h (Figure 4a). After 6 h, multiple fluorescence signals by L-EVs were observed within the upper layer of the dermis. C-EVs showed relatively low fluorescence signals compared to L-EVs in the epidermis and dermis of skin tissue at all times (Figure 4b). According to results of the DiO-signal observed in cells of the dermis layer, L-EVs showed an improved green signal in cells of the dermis layer by 238% compared to C-EVs (Figure 4c).

In order to analyze the changes in ECM synthesis caused by EVs, the expression of PIP, MMP-1, and TIMP-1 were quantified in order to assess capabilities in collagen synthesis, collagen degradation and MMP inhibition respectively (Figure 4e–g). L-EVs enhanced the expression of PIP and TIMP-1 by 32% and 107% compared to negative control levels, and decreased the expression of MMP-1 by 29% compared to negative control levels. In contrast, C-EVs increased the expression of PIP and TIMP-1 by 11% and 42% compared to negative control levels, and decreased the expression of MMP-1 by 15% compared to negative control levels. Based on the results showing that 10 µg/mL of hLF by itself was not effective for ECM synthesis in skin tissue model, we can conclude that the ECM synthesis efficacy of L-EVs in the tissue was stronger than that of C-EVs.

## 3. Discussion

In this study, we confirmed for the first time that hLF induces a change in intracellular Ca^2+^ levels in hADSCs and thereby increases hADSC-derived EV biogenesis. A recent study investigated the alteration of neutrophil calcium levels by using hLF in media containing Ca^2+^ [29]. According to the authors, the addition of hLF to neutrophils resulted in transient calcium channel activation via tyrosine kinase and phospholipase C, and the activation of calcium channels by hLF was confirmed to be enhanced by the addition of CaCl_2_. In our study, the effect of hLF-supplemented CaCl_2_ on hADSCs was analyzed. Initially, no changes were observed in the intracellular Ca^2+^ levels of hADSCs on day 1 even after the addition of hLF to basal media. However, when hLF and CaCl_2_ were administered together, the increased intracellular Ca^2+^ level was observed in hADSCs and maintained for longer than a day. Furthermore, the amount of EVs markers in hADSC-CM increased in proportion to the concentration of CaCl_2_. Therefore, hLF appears to directly affect hADSC-derived EV biogenesis by increasing the intracellular Ca^2+^ levels of hADSCs.

Generally speaking, stimulants that are used to increase the secretion of EVs change both their internal and external characteristics [14]. Many pre-existing stimulants were substances that were unnecessary or even detrimental as components for EVs in their functionality. However, hLF is a multi-functional protein that has cell-beneficial functions such as dermal fibroblast proliferation and ECM synthesis [27]. It is also known that hADSC-derived EVs enhance cell proliferation and ECM synthesis in dermal fibroblasts [10,36]. Due to the fact that hLF and hADSC-derived EVs both positively effects the cells in terms of proliferation and ECM synthesis, we expect that their synergy will result in a stronger ECM synthesis and proliferation effects on dermal fibroblasts when compared to the effects of stem cell-derived EVs alone. The Manoj Raje group discovered that hLF bound to EVs of a macrophage cell line when hLF and EVs were mixed together [32]. This group suggested that the GAPDH of the EVs acts as a hLF binding site, and found that macrophage EVs can act as a useful carrier for delivering hLF to other cells. In this study, we discovered that hLF could also be used in the process of culturing hADSCs to stimulate the secretion of EVs, in contrast to adding hLF to the hADSC-derived EVs after isolation. By culturing hADSCs with hLF, hLFs can bind to hADSC-derived EVs and migrate to the dermal fibroblasts along with hADSC-derived EVs. The characteristic change of hADSC-derived EVs did not have a toxic effect on dermal fibroblasts, but rather increased their cellular activities. We need to investigate the altered characteristics of hLF-stimulated EVs in more detail. hLF is not only known to enhance cell proliferation, but also as a multifunctional protein through its inhibition of TGF-β, which prevents cells from differentiation into myofibroblasts and confers anti-EMT (epithelial to mesenchymal transition) properties [27,37,38]. However, based on current results and previously reported data, we cannot rule out the possibility that hLF-bound EVs can induce EMT and myofibroblast differentiation. It seems that a wide-range characteristic change analysis of hLF-stimulated hADSC derived EVs produced with hLF stimulation is imperative in future research efforts to uncover wider ranges of EV applications.

We proved in vitro that hLF-stimulated hADSC-derived EVs increased the activity of dermal fibroblasts through an increase in both of function and number of secreted EVs. Therefore, in order to assess the net effect of hLF on hADSC-derived EVs, hLF-stimulated EVs and unstimulated EVs were isolated from CMs that were derived from the same number of hADSCs, and then were applied to a skin tissue model to compare their ability in tissue rejuvenation. The epidermis is one of the more difficult tissue barriers to permeate through. EVs have been reported to have higher skin permeability compared to other solutes with hydrophilic properties [9,39,40]. In this study, our group demonstrated that hADSC-derived EVs increased ECM synthesis in a skin tissue model, and that the increased number of hADSC-derived EVs by hLF stimulation exhibited enhanced efficacy through increased permeation.

## 4. Materials and Methods

### 4.1. hADSC Isolation and Culture

Human adipose tissue was harvested from a 17-year-old man who had undergone liposuction surgery. The patient provided his written informed consent. The study was approved by the Ethics Review Committee of the Institutional Review Board at Hallym University Medical Center (IRB no. 2017-118). The tissues were washed and enzymatically digested with 0.075% (*w*/*v*) collagenase of type Ⅱ (Sigma–Aldrich, St. Louis, MO, USA) under gentle agitation at 37 °C for 40 min. Isolated hADSCs were cultured at a density of 4.5 × 10^3^ cells/cm^2^ in expansion media containing α-minimum essential media (α-MEM; Welgene, Gyeongsang, Korea), 10% fetal bovine serum (FBS; Capricorn Scientific GmbH, Ebsdorfergrund, Germany), and 5 ng/mL of recombinant human basic fibroblast growth factor (bFGF; R&D system, Minneapolis, MN, USA). The media were refreshed every 2 days, and the cells were sub-cultured at 80% confluency up to six passages.

### 4.2. Preparation of hADSC-Conditioned Media

hADSCs were seeded on culture plates at a density of 3 × 10^3^ cells/cm^2^ and cultured in expansion media for 3 days. To remove the EVs generated from hADSCs during the expansion period, the cells were rinsed with phosphate-buffered saline (PBS; Capricorn Scientific GmbH, Ebsdorfergrund, Germany) once and further cultured in α-MEM without serum for 24 h. After washing the cells with PBS, they were incubated with α-MEM containing 10% particle-depleted serum replacement (SR; XenoFree CTS; Gibco Life Technologies, Carlsbad, CA, USA) with varying concentrations of human lactoferrin (Aspira Scientific, Oakland, CA, USA) and CaCl_2_ (Sigma–Aldrich, St Louis, MO, USA). After 2 days, the conditioned media (CM) were harvested and the remaining cells were detached for future analysis.

The Ca^2+^ concentration in the hADSCs cultured by the method detailed above was investigated. The calcium contents in the hADSCs were measured using a Calcium Colorimetric Assay kit (BioVision, Milpitas, CA, USA). The cells (1 × 10^5^) were briefly lysed in a calcium assay buffer before being added to 96-well plates. Then, the chromogenic reagent and the calcium assay buffer were added to each well. After incubation for 10 min at room temperature, the absorbance was measured at 575 nm.

The activity of the hADSCs was measured by using an MTT assay. The cells were reacted with a 0.5-mg/mL MTT (Sigma–Aldrich, St. Louis, MO, USA) reagent for 2 h at 37 °C in a 5% CO_2_ chamber. After removing the agent, an equal volume of isopropyl alcohol (Sigma–Aldrich, St. Louis, MO, USA) was added and shaken at room temperature for 10 min. The solutions were measured under a detection wavelength of 570 nm using an ELISA reader (Versamax, Molecular device, San Jose, CA, USA).

The CD9 concentration in the hADSC-CM was investigated using a double sandwich enzyme-linked immunosorbent assay (ELISA) using a human CD9 ELISA kit (MY BioSource, Inc., San Diego, CA, USA). All steps were conducted in accordance with the manufacturer’s instructions, and then the absorbance was measured.

### 4.3. EV Separation & Characterization

Particle-depleted SR and a concentrated CM were obtained using filtration spin columns (Vivaspin 20; Sartorius AG, Goettingen, Germany) with a 100-kDa molecular weight cut-off. Briefly, either SR or CM was placed in the columns and centrifuged until 90% of the media had passed through the membrane. The particle-depleted SR was collected at the lower part of the column. The concentrated CM was collected at the upper part of the column.

EVs were separated from the concentrated CM using qEVoriginal (IZONscience, Christchurch, New Zealand) following the manufacturer’s protocol. The concentrated CM was placed onto the loading frit, and when the entire volume of CM was loaded completely, PBS was added to the loading frit and the void volume was collected.

Particle size distribution and the number of EVs were identified via nanoparticle tracking analysis (NTA) using NTA software (NS300; Malvern Instruments, Malvern, UK) equipped with a 488-nm laser. EVs were diluted with PBS until the appropriate number was detected following NTA instructions.

To observe the morphology of EVs by TEM, separated EVs (50 µL) were placed on formvar-carbon coated mesh 400 grids for 30 min to allow for adherence, then fixed in 4% paraformaldehyde for 10 min. To visualize the hLFs bound to EVs, the fixed EVs on grids were blocked with 0.4% BSA/PBS for 1 h. The EVs were incubated with a 1/20 dilution anti-lactoferrin antibody (Abcam Inc., Toronto, ON, Canada) and then incubated with a 1/400 dilution of a gold-labeled secondary antibody. After fixing the EVs once more, the grids were then transferred into the wash buffer contained in the Exosome-TEM-easy kit (101bio, Palo Alto, CA, USA). EVs on grids were negatively stained with an EM solution for 10 min and then viewed under TEM (FEI Company, Hillsboro, OR, USA) analysis at a voltage of 120 kV.

For the analysis of EVs markers, ADSCs and EVs lysate was prepared using RIPA buffer (EMD millipore Corp., Billerica, MA, USA) and measured by BCA assay (iNtRON biotechnology, Gyeonggi, Korea). The protein which was denatured using 5×laemmli’s sample buffer (Elpis biotech, Daejeon, Korea) was separated via SDS-PAGE gel and then transferred to a nitrocellulose membrane (Whatman GmbH, Dassel, Germany). The nitrocellulose membrane was stained with ponceau S (Amresco., solon, OH, USA). For western blot, the membrane was blocked with 5% skimmed milk in PBS-T and then incubated with primary antibodies, such as rabbit anti-CD9 (Cell Signaling Technology, Danvers, MA, USA), mouse anti-CD81 (Santa Cruz Biotechnology, Santa Cruz, CA, USA), mouse anti-calnexin (Santa Cruz Biotechnology, Santa Cruz, CA, USA), or β-actin (Sigma–Aldrich, St. Louis, MO, USA) overnight at 4 °C. After washing with PBS-T, the membrane was incubated with the following secondary antibodies, goat anti-rabbit antibody (Biorad, Hercules, CA, USA), or mouse IgG kappa binding protein (Santa Cruz Biotechnology, Santa Cruz, CA, USA), for 1 h at room temperature and then reacted with ECL solution (Cytiva, Marlborough, MA, USA). Images were visualized with a luminescent image analyzer (Fujifilm, Tokyo, Japan). The bands were quantitatively measured using Image-J software (National Institute for Health, Bethesda, MD, USA).

The levels of hLF and calcium of EVs obtained after EV separation were measured by using a human lactoferrin ELISA kit (Novus biologicals, Centennial, CO, USA). All steps were conducted in accordance with the manufacturer’s instructions and then absorbance was measured.

### 4.4. Effects of hADSCs-EVs on 2-Dimensional (2D) HDFs

HDFs were obtained from Biosolution Co., Ltd., Seoul, Korea. The cells were seeded at a density of 5 × 10^3^ cells/well in a 48-well plate and cultured in fibroblast growth media-2 Bullet kit (FGM-2; Lonza, Walkersville, MD, USA) for 24 h. To assess the effect of the hADSCs-EVs on HDFs, hADSC-EVs were diluted in FGM-2 and treated to HDFs for 48 h and compared to HDFs cultured in FGM-2 as a negative control. The activity of the hADSC-EVs on HDFs was analyzed using an MTT assay as described in the above process.

To observe the transfer of hLF along with EVs to HDFs, the membrane of hLF-bound EVs was stained with both a DiO and a lactoferrin antibody. A 0.5-μL aliquot of DiO (Invitrogen, Carlsbad, CA, USA) per 1 mL of EVs was diluted and incubated at room temperature for 30 min. EVs were incubated in a 1/20 dilution of anti-lactoferrin antibody at room temperature for 1 h and then stained with a 1/1000 dilution of AlexaFluor 555^TM^ anti-rabbit antibody (Thermo Fisher Scientific, Waltham, MA, USA) for 2 h. After separating EVs using the qEVoriginal kit, the number of DiO-labeled EVs was counted using NTA with a green fluorescence filter (500 long pass filter). The stained EVs were then diluted in α-MEM and exposed to 1 × 10^4^ HDF cells that were seeded in 6 well-plate. HDF cells were stained by CellTracker^TM^ Blue CMAC Dye (Invitrogen, Carlsbad, CA, USA) at 37 °C for 30 min before the treatment of EVs. After 24 h, EVs-treated HDFs were washed and observed by Lionheart FX (BioTek, Santa Barbara, CA, USA).

### 4.5. Effects of hADSCs-EVs on 3-Dimensional (3D) Skin Model

To analyze the effect of EVs in a 3D-skin tissue model, a reconstructed full-thickness human skin model (KeraSkin™-FT) was obtained from Biosolution Co., Ltd., Seoul, Korea. KeraSkin™-FT was cultured on a 6-well plate containing tissue culture media (Welgene, Gyeongsang, Korea). The DiO-stained EVs were then applied to KeraSkin™-FT for 1 to 6 h, then analyzed.

To visualize the skin permeation capability of the EVs, KeraSkin™-FT models were fixed in 4% formaldehyde (Sigma-Aldrich, St. Louis, MO, USA) and embedded in paraffin. Subsequently, the tissue was cut vertically into 5-µm thick sections and attached to a glass slide (Paul marienfeld GmbH & Co. KG, Lauda-Königshofen, Germany). The tissues were counterstained by DAPI (VECTASHIELD; vector laboratories Inc., Burlingame, CA, USA) and observed under fluorescence microscopy (DM4000B; Leica, Wetzlar, Germany). For histological analysis, thin sections of the tissue were stained with hematoxylin (Merck KGaA, Darmstadt, Germany) and eosin (H&E) (Sigma-Aldrich, St. Louis, MO, USA) and observed under a light microscope (Olympus Corporation, Tokyo, Japan).

To evaluate ECM synthesis, the tissue culture media of KeraSkin™-FT were separated from the 6-well plate. Pro-collagen type 1C-PEPTIDE ELISA kit (PIP; Takara Bio Inc., Shiga, Japan), TIMP-1 ELISA kit (R&D system, Minneapolis, MN, USA), and MMP-1 ELISA kit (R&D system, Minneapolis, MN, USA) were used for quantification of their respective proteins in the media.

### 4.6. Statistical Analysis

Statical analysis of all data was carried out using Prism Version 5.01 software (GraphPad prism software, San Diego, CA, USA). For comparisons between two groups, two-tail Student’s unpaired *t*-test was used. For analyses among more than two groups, one-way ANOVA following Tukey’s post hoc test was used. Statistical results of *p* < 0.05 were considered to be statistically significant differences.

## 5. Conclusions

We proved that hLF is a suitable stimulant for the enhanced production of hADSC-derived EVs in regenerative applications. This study is meaningful in that it suggests a method to increase the efficacy of hADSC-derived EVs by changing nothing but the hADSC-culture conditions. The results of this study should provide new insights into the development and application of stem cell-derived EVs for various tissue regeneration and repair fields.

## 6. Patents

J.K., G.E.Y., and J.L. filed and licensed patent application on hADSC-conditioned media stimulated by hLF.

## Data Availability

The datasets used and/or analyzed during the current study are available from the corresponding author upon reasonable request.
